# Correction: Hao et al. Effects of Ten Different Exercise Interventions on Motor Function in Parkinson’s Disease Patients—A Network Meta-Analysis of Randomized Controlled Trials. *Brain Sci.* 2022, *12*, 698

**DOI:** 10.3390/brainsci13060885

**Published:** 2023-05-31

**Authors:** Zikang Hao, Xiaodan Zhang, Ping Chen

**Affiliations:** Department of Physical Education, Laoshan Campus, Ocean University of China, 238 Song Ling Rd., Qingdao 266100, China; haozikang@stu.ouc.edu.cn (Z.H.); zhangxiaodan@stu.ouc.edu.cn (X.Z.)

## Text Correction

There was an error in the original article [1]. A part of the *Brain Sciences* journal’s template text was left in the “Materials and Methods” section by mistake.

A correction has been made to the Materials and Methods section, Section number 2.6, and the second and the third paragraph. The redundant text from the instruction template was removed. Please find the corrected Section 2.6. below:

*2.6. Risk of Bias of Individual Studies*

“Two researchers independently assessed the risk of bias (ROB), in accordance with the Cochrane Handbook version 5.1.0 tool for assessing ROB in RCTs. The following seven domains were considered: (1) randomized sequence generation, (2) treatment allocation concealment, blinding of (3) participants and (4) personnel, (5) incomplete outcome data, (6) selective reporting, and (7) other sources of bias. Trials were categorized into three levels of ROB by the number of components for which high ROB potentially existed: high risk (five or more), moderate risk (three or four), and low risk (two or less) [16]”.

Please find the part of the template text that has been removed below:

“Research manuscripts reporting large datasets that are deposited in a publicly available database should specify where the data have been deposited and provide the relevant accession numbers. If the accession numbers have not yet been obtained at the time of submission, please state that they will be provided during review. They must be provided prior to publication.

Interventionary studies involving animals or humans, and other studies that require ethical approval, must list the authority that provided approval and the corresponding ethical approval code”.

The authors apologize for any inconvenience caused and state that the scientific conclusions are unaffected. The original article has been updated.
