# Supplementary material for: Effects of Ten Different Exercise Interventions on Motor Function in Parkinson’s Disease Patients—A Network Meta-Analysis of Randomized Controlled Trials
Source: Brain Sci. 2022 May 27;12(6):698. doi: 10.3390/brainsci12060698 (PMC9221238; doi:10.3390/brainsci12060698)
Supplement: Supplementary file 1 [file brainsci-12-00698-s001.zip › brainsci-1731259-supplementary.pdf]

Supplementary Table S1. Risk of bias for each included studies.

Study	Random sequence generation	Allocation concealment	Blinding of participants and personnel	Blinding of outcome assessment	Incomplete outcome data	Selective reporting	Other bias	Overall Score (Category)
Vivas	Low	Unclear	Unclear	Unclear	High	Low	Low	4 （moderate）
Carroll	Low	Low	High	Unclear	High	Low	Low	3 （moderate）
Volpe	Low	Low	High	Low	Low	Low	Low	1 （low）
Volpe	Low	Low	Low	Unclear	Low	Low	Low	1 （low）
Perez-de	Low	Unclear	Low	Unclear	Low	Low	Low	2 （low）
Kurt	Low	Unclear	Low	Unclear	Low	Low	Low	2 （low）
Wang	Low	Unclear	Low	Unclear	Low	Low	Low	2 （low）
Palamara	Low	Low	Low	Unclear	Low	Low	Low	1 （low）
Clerici	Low	Low	Unclear	Unclear	High	Low	Low	3 （moderate）
Yuan	Unclear	Low	High	High	Low	Low	Unclear	4 （moderate）
Pazzaglia	Low	Low	High	High	Low	Low	Unclear	3

								（moderate）
Xia	Low	Unclear	High	Unclear	Low	Low	High	4（moderate）
Santos	High	Unclear	High	Unclear	Low	Low	Unclear	5（high）
Tollar	Low	Low	High	High	Low	Low	Low	2（low）
Song	Low	Low	High	Unclear	High	Low	Low	3（moderate）
Lee	Unclear	Unclear	High	Unclear	Unclear	Low	Low	5（high）
Li	Low	Unclear	Unclear	Low	Low	Low	Unclear	3（moderate）
Vieira	Unclear	Unclear	High	Low	Low	Unclear	Unclear	5（high）
De lima	Low	Low	High	Low	Low	Unclear	Unclear	3（moderate）
Kwok	Low	Low	High	Low	Low	Low	Unclear	2（low）
Leal	Low	Low	High	Low	Low	Unclear	Unclear	3（moderate）
Schlenstedt	Low	Low	High	Low	Low	Unclear	Unclear	3（moderate）
Tang	Low	Unclear	Unclear	Unclear	Low	Unclear	Unclear	5（high）
Choi	Unclear	Unclear	Unclear	Low	Low	Low	Unclear	4

								（moderate）
Amano	Unclear	Unclear	High	Low	Low	Low	Low	3 （moderate）
Hackney	Low	Low	Low	Low	Low	Unclear	Unclear	2 （low）
Vergara-Diaz	Low	Low	High	Low	Low	Low	Unclear	2 （low）
Li	Low	Unclear	Unclear	Low	Low	Low	Unclear	3 （moderate）
Choi	Unclear	Unclear	Unclear	Low	Unclear	Low	Unclear	5 （high）
Gao	Low	Unclear	High	Low	Low	Low	Unclear	3 （moderate）
You	Low	Low	High	Low	Low	Low	Unclear	2 （low）
Xiao	Low	Unclear	Unclear	Low	Low	Low	Unclear	3 （moderate）
Shi	Unclear	Unclear	Unclear	Low	Unclear	Low	Unclear	5 （high）
Wang	Low	Low	High	Unclear	High	Low	Unclear	4 （moderate）
Marieke	Unclear	Unclear	High	Low	Low	Low	Low	3 （moderate）
Cheung	Low	Unclear	Unclear	Low	Low	Low	Unclear	3 （moderate）
Kwok	Low	Low	High	Low	Low	Low	Unclear	1

								（low）
Khuzema	Low	Low	Low	High	Low	Low	Unclear	2（low）
Ni	Low	Low	Unclear	High	Low	Low	Unclear	3（moderate）
Sharma	Unclear	Unclear	Unclear	Unclear	Unclear	Low	Unclear	6（high）
Song	Low	Low	High	Low	Low	Low	Unclear	2（low）
Michels	Low	Low	Unclear	Low	Low	Unclear	Unclear	3（moderate）
Volpe	Low	Low	High	Low	Unclear	Low	Unclear	3（moderate）
Shanahan	Unclear	Low	Low	Low	Unclear	Low	Unclear	3（moderate）
Hackney	Low	Low	Low	Low	Low	Unclear	Unclear	2（low）
Rawson	High	Unclear	Unclear	Low	Low	Low	Unclear	4（moderate）
Duncan	Low	Low	Unclear	Low	Low	Low	Unclear	2（low）
Solla	Low	Low	High	High	Low	Low	Unclear	3（moderate）
Romenets	Low	Low	High	High	Low	Low	Unclear	3（moderate）
Shulman	Low	Low	High	Low	Low	Low	Unclear	2

								（low）
Carvalho	Unclear	Unclear	High	Low	Low	Low	Low	3（moderate）
Sage	Unclear	Unclear	Unclear	Low	Low	Low	Unclear	4（moderate）
Cugusia	Unclear	Unclear	Unclear	Unclear	Unclear	Low	Low	5（high）
Bang	Unclear	Low	High	Low	Low	Low	Unclear	3（moderate）
Bello	Unclear	Unclear	Unclear	Unclear	Unclear	Unclear	Unclear	7（high）
Kolk	Low	Low	Low	Low	Low	Low	Unclear	1（low）
Sacheli	Unclear	Unclear	High	Low	Low	Low	Unclear	2（low）
Ridgel	Unclear	Unclear	Unclear	Unclear	Low	Low	Unclear	5（high）
Arcolin	Unclear	Unclear	Unclear	Unclear	Low	Low	Unclear	5（high）
Tollar	Low	Low	High	Low	Low	Unclear	Unclear	3（moderate）

Supplementary Table S2. Consistency test for UPDRS.

	Coef.	Std. Err.	z	P>|z|	[95% Conf. Interval]	
B VS CON	-.1622021	1.781536	-0.09	0.927	-3.653948	3.329544
C VS CON	1.531016	1.216439	1.26	0.208	-.8531614	3.915193
D VS CON	2.932478	1.249295	2.35	0.109	.4839043	5.381052
E VS CON	-.7685721	1.963292	-0.39	0.695	-4.616555	3.07941
F VS CON	-1.968178	1.849176	-1.06	0.287	-5.592496	1.656141
G VS CON	.9447259	2.08785	0.45	0.651	-3.147385	5.036837
H VS CON	1.234108	1.986855	0.62	0.535	-2.660056	5.128273
I VS CON	-1.326081	1.695263	-0.78	0.434	-4.648735	1.996574
J VS CON	-1.188614	2.062386	-0.58	0.564	-5.230816	2.853589
K VS CON	-1.85778	2.500079	-0.74	0.457	-6.757844	3.042284
L VS CON	-1.579226	2.125342	-0.74	0.457	-5.744821	2.586368

Supplementary Table S3. Consistency test for TUGT.

	Coef.	Std. Err.	z	P>|z|	[95% Conf. Interval]	
B VS CON	1.568637	2.208094	0.71	0.477	-2.759149	5.896422
C VS CON	1.776689	.693302	2.56	0.100	.4178419	3.135536
D VS CON	1.668695	.8337112	2.00	0.075	.0346509	3.302739
E VS CON	.789449	1.312764	0.60	0.548	-1.783521	3.362419
F VS CON	.4265893	1.046139	0.41	0.683	-1.623804	2.476983
G VS CON	-.5216772	.9407811	-0.55	0.579	-2.365574	1.32222
H VS CON	.1040121	.9621334	0.11	0.914	-1.781735	1.989759
I VS CON	.2529556	1.113036	0.23	0.820	-1.928555	2.434467
J VS CON	.3596071	1.270027	0.28	0.777	-2.129601	2.848815
K VS CON	.6015271	1.239753	0.49	0.628	-1.828345	3.031399
L VS CON	-.7282505	1.192294	-0.61	0.541	-3.065103	1.608602

Supplementary Table S4. Consistency test for BBS.

	Coef.	Std. Err.	z	P>|z|	[95% Conf. Interval]	
B VS CON	3.287298	2.856736	1.15	0.250	-2.311802	8.886398
C VS CON	-2.221698	1.518548	-1.46	0.143	-5.197996	.7546014
D VS CON	-.958803	2.031787	-0.47	0.637	-4.941032	3.023426
E VS CON	1.400826	2.918556	0.48	0.631	-4.319439	7.12109
F VS CON	4.851568	2.659363	1.82	0.068	-.3606886	10.06382
G VS CON	3.941234	3.523446	1.12	0.263	-2.964593	10.84706
H VS CON	2.540235	2.723636	0.93	0.351	-2.797993	7.878464
I VS CON	-.2192184	3.563543	-0.06	0.951	-7.203634	6.765197
J VS CON	2.643499	2.438002	1.08	0.278	-2.134897	7.421895
K VS CON	2.357567	3.8503	0.61	0.540	-5.188883	9.904016
L VS CON	2.484526	2.88599	0.86	0.389	-3.17191	8.140963
